# *N*-(4-Chloro-2-nitro­phen­yl)methane­sulfonamide

**DOI:** 10.1107/S1600536808031048

**Published:** 2008-09-30

**Authors:** Muhammad Zia-ur-Rehman, Jamil Anwar Choudary, Nosheen Akbar, Islam Ullah Khan, Muhammad Nadeem Arshad

**Affiliations:** a Applied Chemistry Research Centre, PCSIR Laboratories Complex, Lahore 54600, Pakistan; b Institute of Chemistry, University of the Punjab, Lahore 54590, Pakistan; c Centre for High Energy Physics, University of the Punjab, Lahore 54590, Pakistan; d Department of Chemistry, Government College University, Lahore 54000, Pakistan

## Abstract

The title compound, C_7_H_7_ClN_2_O_4_S, is of inter­est as a precursor to biologically active substituted quinolines. Its structure resembles those of the previously reported *N*-phenyl­methane sulfonamide and its 4-nitro, 4-fluoro and 4-bromo derivatives, with slightly different geometric parameters. An intra­molecular N—H⋯O hydrogen bond gives rise to a six-membered ring. Inter­molecular C—H⋯O contacts stabilize the crystal packing.

## Related literature

For related literature, see: Ahn *et al.* (1997 ▶); Allen *et al.* (1987 ▶); Ozbek *et al.* (2007 ▶); Siddiqui *et al.* (2007 ▶); Gennarti *et al.* (1994 ▶); Gowda *et al.* (2007*a* ▶,*b* ▶,*c* ▶); Hanson *et al.* (1999 ▶); Moree *et al.* (1991 ▶); Oppolzer *et al.* (1991 ▶); Rough *et al.* (1998 ▶); Zia-ur-Rehman *et al.* (2005 ▶, 2006 ▶, 2007 ▶, 2008 ▶).
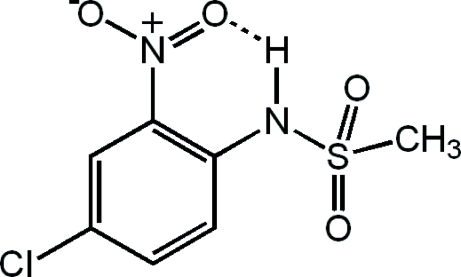


## Experimental

### 

#### Crystal data

C_7_H_7_ClN_2_O_4_S
*M* *_r_* = 250.67
Monoclinic, 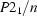

*a* = 11.728 (3) Å
*b* = 4.9798 (13) Å
*c* = 17.988 (5) Å
β = 107.334 (8)°
*V* = 1002.8 (5) Å^3^
*Z* = 4
Mo *K*α radiation
μ = 0.58 mm^−1^
*T* = 296 (2) K
0.22 × 0.14 × 0.07 mm

#### Data collection

Bruker APEXII CCD area-detector diffractometer
Absorption correction: none
10700 measured reflections
2590 independent reflections
1199 reflections with *I* > 2σ(*I*)
*R*_int_ = 0.081

#### Refinement

*R*[*F* ^2^ > 2σ(*F* ^2^)] = 0.050
*wR*(*F* ^2^) = 0.147
*S* = 0.97
2556 reflections
140 parameters
H atoms treated by a mixture of independent and constrained refinement
Δρ_max_ = 0.25 e Å^−3^
Δρ_min_ = −0.39 e Å^−3^

### 

Data collection: *APEX2* (Bruker, 2007 ▶); cell refinement: *SAINT* (Bruker, 2007 ▶); data reduction: *SAINT*; program(s) used to solve structure: *SHELXS97* (Sheldrick, 2008 ▶); program(s) used to refine structure: *SHELXL97* (Sheldrick, 2008 ▶); molecular graphics: *SHELXTL* (Sheldrick, 2008 ▶); software used to prepare material for publication: *WinGX* (Farrugia, 1999 ▶) and *PLATON* (Spek, 2003 ▶).

## Supplementary Material

Crystal structure: contains datablocks I, global. DOI: 10.1107/S1600536808031048/bt2794sup1.cif

Structure factors: contains datablocks I. DOI: 10.1107/S1600536808031048/bt2794Isup2.hkl

Additional supplementary materials:  crystallographic information; 3D view; checkCIF report

## Figures and Tables

**Table 1:** Hydrogen-bond geometry (Å, °)

*D*—H⋯*A*	*D*—H	H⋯*A*	*D*⋯*A*	*D*—H⋯*A*
N1—H1⋯O3	0.80 (4)	2.03 (4)	2.631 (4)	131 (3)
C3—H3⋯O3^i^	0.93	2.59	3.417 (4)	148
C5—H5⋯O2^ii^	0.93	2.47	3.325 (5)	152
C6—H6⋯O2	0.93	2.27	2.951 (5)	130
C7—H8⋯O3^iii^	0.96	2.53	3.394 (5)	150

Symmetry codes: (i) 
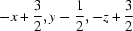
; (ii) 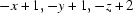; (iii) 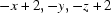.

**Fig. 1.**
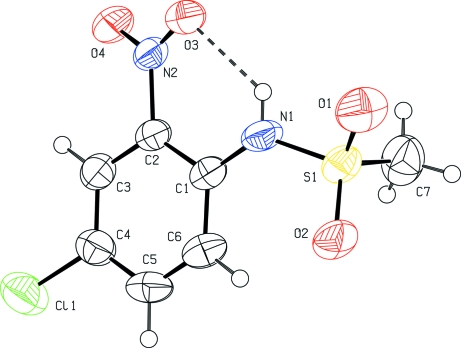
The asymmetric unit of the title compound. Displacement ellipsoids are drawn at the 50% probability level.

**Fig. 2.**
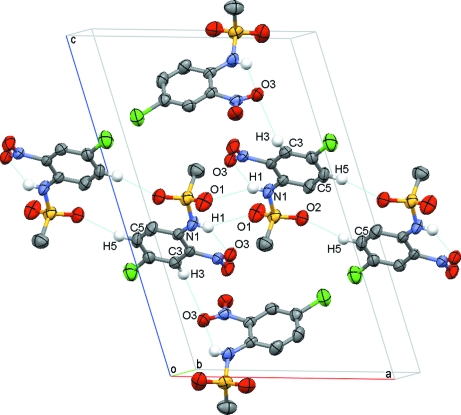
Perspective view of the crystal packing showing hydrogen bonds (dashed lines). H atoms not involved in hydrogen bonding have been omitted for clarity.

## supplementary crystallographic information

### Comment

Sulfonamides are familiar for their enormous potential as biologically active molecules (Hanson *et al.*, 1999; Moree *et al.*, 1991; Rough *et al.*, 1998). They are being used as anti-microbial (Ozbek *et al.*, 2007), anti-convulsant (Siddiqui *et al.*, 2007), and for the treatment of inflammatory rheumatic and non-rheumatic processes including onsets and traumatologic lesions (Gennarti *et al.*, 1994). Besides, these are known as compounds being used as agricultural agents and chiral auxiliaries (Ahn *et al.*, 1997; Oppolzer *et al.*, 1991). Among these, alkyl sulfonanilides are of special interest due to their stereochemistry with amide hydrogen on one side of the plane of benzene ring making it a good receptor site for biological reactions. In the present paper, the structure of *N*-(4-chloro-2-nitrophenyl)methanesulfonamide has been determined as part of a research program involving the synthesis and biological evaluation of sulfur containing heterocyclic compounds (Zia-ur-Rehman *et al.*, 2005, 2006, 2007, 2008). In the molecule of (I) (Fig. 1), bond lengths and bond angles are almost similar to those in the related molecules (Gowda *et al.*, 2007*a*,*b*,*c*) and are within normal ranges (Allen *et al.*, 1987). Intramolecular interaction [N1—H1···O3] is observed in the title molecule giving rise to six-membered hydrogen bonded ring. Each molecule is centrosymmetrically linked to its adjacent one through intermolecular [N1—H1···O1] hydrogen bonds on one side, and *via* [C5—H5···O2] hydrogen bonds on the other side, giving rise to a zigzag chain along *a* axis. Each molecule of a chain is further linked to the member of adjacent chain *via* [C3—H3···O3] hydrogen bonds along *c* giving rise to a three dimensional network.

### Experimental

A mixture of 4-chloro-2-nitroaniline (3.452 g; 20.0 mmoles) and mesyl chloride (2.52 g; 22.0 mmoles) and toluene (25.0 ml) was heated to reflux for half an hour. Solvent was then distilled off under reduced pressure and the resultant solids were washed with cold methanol. Crystals suitable for analysis were obtained by slow evaporation of methanolic solution over a period of two days.

### Refinement

H atoms bound to C were placed in calculated positions (C—H distance = 0.95 Å) using a riding model. H atoms on N and O were freely refined.

### Crystal data

**Table:**

C_7_H_7_ClN_2_O_4_S	*F*(000) = 512
*M**_r_* = 250.67	*D*_x_ = 1.660 Mg m^−^^3^
Monoclinic, *P*2_1_/*n*	Melting point: 388 K
Hall symbol: -P 2yn	Mo *K*α radiation, λ = 0.71073 Å
*a* = 11.728 (3) Å	Cell parameters from 1283 reflections
*b* = 4.9798 (13) Å	θ = 2.4–20.9°
*c* = 17.988 (5) Å	µ = 0.58 mm^−^^1^
β = 107.334 (8)°	*T* = 296 K
*V* = 1002.8 (5) Å^3^	Needle, light yellow
*Z* = 4	0.22 × 0.14 × 0.07 mm

### Data collection

**Table:**

Bruker APEXII CCD area-detector diffractometer	1199 reflections with *I* > 2σ(*I*)
Radiation source: fine-focus sealed tube	*R*_int_ = 0.081
graphite	θ_max_ = 28.7°, θ_min_ = 1.9°
Detector resolution: 7.5 pixels mm^-1^	*h* = −15→15
φ and ω scans	*k* = −6→6
10700 measured reflections	*l* = −23→24
2590 independent reflections	

### Refinement

**Table:**

Refinement on *F*^2^	Primary atom site location: structure-invariant direct methods
Least-squares matrix: full	Secondary atom site location: difference Fourier map
*R*[*F*^2^ > 2σ(*F*^2^)] = 0.050	Hydrogen site location: inferred from neighbouring sites
*wR*(*F*^2^) = 0.148	H atoms treated by a mixture of independent and constrained refinement
*S* = 0.97	*w* = 1/[σ^2^(*F*_o_^2^) + (0.0627*P*)^2^] where *P* = (*F*_o_^2^ + 2*F*_c_^2^)/3
2556 reflections	(Δ/σ)_max_ < 0.001
140 parameters	Δρ_max_ = 0.25 e Å^−^^3^
0 restraints	Δρ_min_ = −0.39 e Å^−^^3^

### Special details

**Table:**

Geometry. All e.s.d.'s (except the e.s.d. in the dihedral angle between two l.s. planes) are estimated using the full covariance matrix. The cell e.s.d.'s are taken into account individually in the estimation of e.s.d.'s in distances, angles and torsion angles; correlations between e.s.d.'s in cell parameters are only used when they are defined by crystal symmetry. An approximate (isotropic) treatment of cell e.s.d.'s is used for estimating e.s.d.'s involving l.s. planes.
Refinement. Refinement of *F*^2^ against ALL reflections. The weighted *R*-factor *wR* and goodness of fit *S* are based on *F*^2^, conventional *R*-factors *R* are based on *F*, with *F* set to zero for negative *F*^2^. The threshold expression of *F*^2^ > σ(*F*^2^) is used only for calculating *R*-factors(gt) *etc*. and is not relevant to the choice of reflections for refinement. *R*-factors based on *F*^2^ are statistically about twice as large as those based on *F*, and *R*- factors based on ALL data will be even larger.

### Fractional atomic coordinates and isotropic or equivalent isotropic displacement parameters (Å^2^)

**Table:**

	*x*	*y*	*z*	*U*_iso_*/*U*_eq_	
Cl1	0.36565 (9)	−0.3010 (2)	0.78879 (7)	0.0757 (4)	
S1	0.82089 (9)	0.53780 (17)	1.02558 (6)	0.0499 (3)	
O1	0.9148 (3)	0.7102 (5)	1.01960 (19)	0.0756 (9)	
O2	0.7141 (2)	0.6562 (5)	1.03188 (16)	0.0647 (8)	
O3	0.8855 (2)	0.1004 (5)	0.85222 (14)	0.0554 (7)	
O4	0.8088 (2)	−0.2740 (5)	0.80406 (15)	0.0613 (7)	
H1	0.852 (3)	0.324 (7)	0.938 (2)	0.047 (11)*	
N1	0.7906 (3)	0.3571 (6)	0.94717 (19)	0.0509 (8)	
N2	0.8034 (3)	−0.0641 (6)	0.83722 (16)	0.0441 (7)	
C1	0.6920 (3)	0.1941 (6)	0.91264 (19)	0.0416 (8)	
C2	0.6953 (3)	−0.0052 (6)	0.85865 (19)	0.0397 (8)	
C3	0.5963 (3)	−0.1573 (7)	0.8216 (2)	0.0473 (9)	
H3	0.6013	−0.2884	0.7859	0.057*	
C4	0.4913 (3)	−0.1147 (7)	0.8376 (2)	0.0507 (9)	
C5	0.4847 (3)	0.0733 (7)	0.8916 (2)	0.0559 (10)	
H5	0.4136	0.0974	0.9037	0.067*	
C6	0.5830 (4)	0.2266 (7)	0.9282 (2)	0.0556 (10)	
H6	0.5767	0.3554	0.9642	0.067*	
C7	0.8741 (4)	0.3148 (8)	1.1025 (3)	0.0756 (13)	
H8	0.9413	0.2183	1.0959	0.113*	
H9	0.8121	0.1903	1.1036	0.113*	
H7	0.8984	0.4127	1.1507	0.113*	

### Atomic displacement parameters (Å^2^)

**Table:**

	*U*^11^	*U*^22^	*U*^33^	*U*^12^	*U*^13^	*U*^23^
Cl1	0.0495 (6)	0.0871 (8)	0.0875 (9)	−0.0078 (5)	0.0156 (6)	−0.0033 (6)
S1	0.0594 (6)	0.0414 (5)	0.0527 (6)	0.0081 (4)	0.0224 (5)	−0.0082 (4)
O1	0.083 (2)	0.0540 (15)	0.100 (2)	−0.0184 (14)	0.0431 (19)	−0.0276 (15)
O2	0.0700 (18)	0.0622 (16)	0.0661 (19)	0.0267 (13)	0.0267 (14)	−0.0096 (13)
O3	0.0468 (15)	0.0678 (16)	0.0571 (18)	−0.0027 (13)	0.0238 (13)	−0.0109 (13)
O4	0.0608 (17)	0.0650 (16)	0.0627 (19)	0.0092 (12)	0.0255 (14)	−0.0258 (13)
N1	0.050 (2)	0.0539 (18)	0.058 (2)	0.0022 (16)	0.0296 (17)	−0.0131 (14)
N2	0.0457 (18)	0.0526 (17)	0.0364 (17)	0.0107 (15)	0.0157 (13)	−0.0016 (13)
C1	0.048 (2)	0.0412 (18)	0.040 (2)	0.0068 (16)	0.0205 (17)	0.0032 (15)
C2	0.042 (2)	0.0445 (18)	0.0361 (19)	0.0112 (15)	0.0167 (16)	0.0071 (14)
C3	0.050 (2)	0.053 (2)	0.040 (2)	0.0066 (17)	0.0149 (17)	−0.0007 (16)
C4	0.042 (2)	0.058 (2)	0.053 (2)	0.0034 (17)	0.0137 (18)	0.0078 (18)
C5	0.044 (2)	0.061 (2)	0.071 (3)	0.0128 (19)	0.029 (2)	0.006 (2)
C6	0.060 (3)	0.053 (2)	0.063 (3)	0.0093 (19)	0.032 (2)	−0.0051 (18)
C7	0.086 (3)	0.075 (3)	0.058 (3)	0.021 (2)	0.010 (2)	0.003 (2)

### Geometric parameters (Å, °)

**Table:**

Cl1—C4	1.741 (4)	C1—C6	1.397 (5)
S1—O2	1.419 (3)	C2—C3	1.380 (5)
S1—O1	1.426 (3)	C3—C4	1.363 (4)
S1—N1	1.621 (3)	C3—H3	0.9300
S1—C7	1.739 (4)	C4—C5	1.368 (5)
O3—N2	1.231 (3)	C5—C6	1.375 (5)
O4—N2	1.215 (3)	C5—H5	0.9300
N1—C1	1.397 (4)	C6—H6	0.9300
N1—H1	0.80 (3)	C7—H8	0.9600
N2—C2	1.461 (4)	C7—H9	0.9600
C1—C2	1.397 (4)	C7—H7	0.9600
			
O2—S1—O1	118.43 (17)	C4—C3—C2	119.7 (3)
O2—S1—N1	109.28 (17)	C4—C3—H3	120.1
O1—S1—N1	104.05 (17)	C2—C3—H3	120.1
O2—S1—C7	108.5 (2)	C3—C4—C5	120.2 (3)
O1—S1—C7	110.0 (2)	C3—C4—Cl1	119.5 (3)
N1—S1—C7	105.77 (19)	C5—C4—Cl1	120.3 (3)
C1—N1—S1	130.3 (3)	C4—C5—C6	120.2 (3)
C1—N1—H1	118 (3)	C4—C5—H5	119.9
S1—N1—H1	108 (3)	C6—C5—H5	119.9
O4—N2—O3	121.9 (3)	C5—C6—C1	121.8 (3)
O4—N2—C2	118.6 (3)	C5—C6—H6	119.1
O3—N2—C2	119.5 (3)	C1—C6—H6	119.1
C2—C1—C6	116.0 (3)	S1—C7—H8	109.5
C2—C1—N1	122.1 (3)	S1—C7—H9	109.5
C6—C1—N1	121.9 (3)	H8—C7—H9	109.5
C3—C2—C1	122.1 (3)	S1—C7—H7	109.5
C3—C2—N2	115.7 (3)	H8—C7—H7	109.5
C1—C2—N2	122.2 (3)	H9—C7—H7	109.5
			
O2—S1—N1—C1	−37.6 (4)	O4—N2—C2—C1	163.9 (3)
O1—S1—N1—C1	−165.0 (3)	O3—N2—C2—C1	−17.0 (4)
C7—S1—N1—C1	79.0 (4)	C1—C2—C3—C4	−0.1 (5)
S1—N1—C1—C2	−161.2 (3)	N2—C2—C3—C4	−179.5 (3)
S1—N1—C1—C6	21.0 (5)	C2—C3—C4—C5	−1.7 (5)
C6—C1—C2—C3	1.3 (5)	C2—C3—C4—Cl1	178.3 (3)
N1—C1—C2—C3	−176.7 (3)	C3—C4—C5—C6	2.2 (6)
C6—C1—C2—N2	−179.3 (3)	Cl1—C4—C5—C6	−177.8 (3)
N1—C1—C2—N2	2.7 (5)	C4—C5—C6—C1	−1.0 (6)
O4—N2—C2—C3	−16.6 (4)	C2—C1—C6—C5	−0.7 (5)
O3—N2—C2—C3	162.5 (3)	N1—C1—C6—C5	177.2 (3)

### Hydrogen-bond geometry (Å, °)

**Table:**

*D*—H···*A*	*D*—H	H···*A*	*D*···*A*	*D*—H···*A*
N1—H1···O3	0.80 (4)	2.03 (4)	2.631 (4)	131 (3)
C3—H3···O3^i^	0.93	2.59	3.417 (4)	148
C5—H5···O2^ii^	0.93	2.47	3.325 (5)	152
C6—H6···O2	0.93	2.27	2.951 (5)	130
C7—H8···O3^iii^	0.96	2.53	3.394 (5)	150

Symmetry codes: (i) −*x*+3/2, *y*−1/2, −*z*+3/2; (ii) −*x*+1, −*y*+1, −*z*+2; (iii) −*x*+2, −*y*, −*z*+2.
